# Supplementary material for: Towards silent and efficient flight by combining bioinspired owl feather serrations with cicada wing geometry
Source: Nat Commun. 2024 May 21;15:4337. doi: 10.1038/s41467-024-48454-3 (PMC11109230; doi:10.1038/s41467-024-48454-3)
Supplement: Supplementary file 1 — Supplementary Information [file 41467_2024_48454_MOESM1_ESM.pdf]

Supplementary Information

Towards silent and efficient flight by combining bioinspired owl feather serrations with cicada wing geometry

Zixiao Wei[1,2], Stanley Wang[1,2], Sean Farris[1], Naga Chennuri[1], Ningping Wang[1], Stara Shinsato[1], Kahraman Demir[1], Maya Horii[1], and Grace X. Gu[1*]

[1] Department of Mechanical Engineering, University of California, Berkeley, CA 94720, USA

[2] These authors contributed equally

[*]Corresponding author: ggu@berkeley.edu

Supplementary Note 1. Acoustic modelling and OASPL calculation

For the acoustics model, we adopt the Ffowcs Williams-Hawkings (FW-H) equation[1] as the governing equation for our analysis, as shown blow:

$$\frac{\partial p'}{\partial t^2} - c^2 \nabla^2 p' = \frac{\partial^2}{\partial x_i \partial x_j}\left[T_{ij} H(f)\right] + \frac{\partial F_i \delta(f)}{\partial x_i} + \frac{\partial Q_i \delta(f)}{\partial t} \tag{1}$$

In this context, p' represents the pressure fluctuation, c signifies the reference sound speed underwater, f denotes the frequency, u_i represents the fluid velocity components within Cartesian coordinates, and u_n corresponds to the velocity component that is normal to the controlled surface. $H(f)$ is the Heaviside step function and $\delta(f)$ is the Dirac-delta function; both are mathematical tools that serve to identify the acoustic surface under investigation. Furthermore, T_{ij}, F_i and Q_i correspond to the Lighthill's stress tensor, surface momentum flux vector, and the surface mass flux vector, respectively. The definition of these tensors are listed as follows:

$$T_{ij} = \rho_0 u_i u_j + p' \delta_{ij} - \tau_{ij} \tag{2}$$

$$F_i = -\left[\rho u_i (u_n - v_n) + \left(p \delta_{ij} - \tau_{ij}\right) n_j\right] \tag{3}$$

$$Q_i = [\rho(u_n - v_n) + \rho_0 v_n] \tag{4}$$

In these equations, v_i represents the velocity component of the controlled source surface, while v_n denotes the corresponding components projected along the surface norm. After solving the pressure and velocity fields, the FW-H model utilizes these data to determine the acoustic pressure, thereby translating the aerodynamically computed fields into acoustic properties. It is noteworthy that the FW-H approach enables dynamic, real-time computation, offering immediate insights and an analytical framework for interpreting the sound propagation within the simulated environment.

In rotor acoustics, the noise is an amalgamation of loading, thickness, and quadrupole contributions, wherein the loading and thickness noises collectively form the dipole noise component. In the acoustic spectrum, both loading and thickness noise present as peak SPL. Broadly speaking, loading noise is related to the variations in the mean pressure term $\bar{p}$ with each revolution, whereas thickness noise is associated with the pressure fluctuation term p'. Within the controlled acoustic source surface, the sound pressures correspond to these sources of noise are mathematically articulated as:

$$p'_T(\mathbf{x}, t) = \frac{1}{4\pi}\left\{\int_{\partial\mathcal{R}} \frac{\rho_0(\dot{v_n} + v_{\dot{n}})}{r(1 - M_r)^2}\, dS + \int_{\partial\mathcal{R}} \frac{\rho_0 v_n\left[r\dot{M}_r + c_0(M_r - M^2)\right]}{r^2(1 - M_r)^3}\, dS\right\} \tag{5}$$

$$p'_L(\mathbf{x},t) = \frac{1}{4\pi}\left\{\frac{1}{c_0}\int_{\partial\mathcal{R}}\left[\frac{\dot{L}_r}{r(1-M_r)^2} + \frac{L_r[r\dot{M} + c_0(M_r - M^2)]}{r^2(1-M_r)^3}\right] dS + \int_{\partial\mathcal{R}}\frac{L_r - L_M}{r^2(1-M_r)^2}\, dS\right\} \tag{6}$$

$$p'_Q(\mathbf{x},t) = p'(\mathbf{x},t) - p'_T(\mathbf{x},t) - p'_L(\mathbf{x},t) \tag{7}$$

In the given context, $p'_T(\mathbf{x},t)$, $p'_L(\mathbf{x},t)$, and $p'_Q(\mathbf{x},t)$ represent the pressure fluctuations associated with thickness noise, loading noise, and quadrupole noises, respectively. The variable r signifies the radial direction. The term M refers to the Mach number, while M_r denotes the Mach number in the radial direction. The radial component of the loading stress, L_r, is defined as $(L_r)_i = L_i r_i$. Lastly, L_M, determined by $(L_M)_i = L_i M_i$, reflects the variation in aerodynamic force attributable to fluid compressibility. Within this framework, thickness and loading noises are quantified as the sound radiation emanating from the acoustic source surface. Specifically, $p'_T(\mathbf{x},t)$ corresponds to the pressure fluctuations resulting from the displacement of air due to the motion of the surface, often described as the air flux transport across the source surface. On the other hand, $p'_L(\mathbf{x},t)$ reflects the pressure fluctuations attributable to aerodynamic loading, which encompasses the forces exerted by airflow over the surface, as well as the temporal rate of change of these forces, commonly referred to as the material derivative of loading.

In terms of the OASPL calculation, the data presented in this manuscript are unweighted to provide insight into the raw sound signals. The A-weighted overall sound pressure level (OASPL) is utilized to encapsulate the entirety of acoustic energy perceived by the human auditory system. This metric accentuates frequencies within the audible spectrum (20 Hz to 20 kHz) and provides a holistic assessment of noise intensity across all resolved frequencies. The A-weighted coefficient for each frequency is computed according to the formula delineated in Eq. (8). Subsequently, the unweighted sound pressure level (SPL) is adjusted using Eq. (9). The determination of OASPL, with respect to a specific set of frequencies and SPLs, is outlined by Eq. (10), which facilitates the calculation of both unweighted and A-weighted OASPL values. It is noteworthy that we calculate the OASPL using a cut-off frequency of 100 Hz to remove the DC bias from the low-frequency bandwidth, which results in a uniform OASPL reduction of approximately 0.1 dB for all designs.

$$R_A(f_i) = SPL(f_i)\cdot\frac{12194^2 f_i^4}{(f_i^2 + 20.6^2)\sqrt{(f_i^2 + 107.7^2)(f_i^2 + 737.9^2)}(f_i^2 + 12194^2)} \tag{8}$$

$$A - SPL(f_i) \approx SPL(f_i) + 20\log_{10}\big(R_A(f)\big) + 2.00 \tag{9}$$

$$OASPL = 10\ \cdot log_{10}\left(\sum_{i=1}^{n} 10^{0.1SPL(f_i)}\right) \tag{10}$$

Supplementary Note 2. Aerodynamic modelling

For enhanced precision in CFD analyses, the selection of an appropriate turbulence model is essential. Our methodology incorporates the Large Eddy Simulation (LES) method for simulating the turbulent flow characteristics, in contrast to the Menter Shear-Stress Transport (SST) model. The SST model integrates blending functions to segregate the far-field and near-wall domains, while it employs the Reynolds Averaged Navier-Stokes (RANS) framework for the calculation of averaged flow properties with additional fluctuating components. In distinction to RANS, the LES methodology is capable of resolving larger scale vortical structures via spatial filtering operations. The sub-grid scale (SGS) model inherent in LES excludes small scales of motion that have a minimal impact on energy, thereby streamlining computational resources toward scales that are more significant dynamically. Specifically, LES concentrates on the scales of energy transfer, which are crucial for accurately predicting the flow's physical behavior. Within LES, the formulation of momentum balance is carefully designed to capture these critical factors, as shown below.

$$\rho\left(\frac{\partial \hat{u}_i}{\partial t}+\frac{\partial \hat{u}_i}{\partial x_j}\hat{u}_j\right)=-\frac{\partial p}{\partial x_i}-\lambda\frac{\partial}{\partial x_i}\left(\frac{\partial \hat{u}_k}{\partial x_k}\right)+\frac{\partial}{\partial x_j}\left(\tau_{ij}^{les}+\tau_{ij}^{sgs}\right) \tag{11}$$

For scales larger than the grid filter, the velocity field undergoes spatial filtering via a convolutional integral, which is mathematically represented by the subsequent equation:

$$\hat{\mathbf{u}}(\mathbf{x},\, t)=\int_{-\infty}^{\infty} G(\mathbf{x}-\boldsymbol{\xi})\,\mathbf{u}(\boldsymbol{\xi},\, t)\; d\xi \tag{12}$$

where $\boldsymbol{x}$ represents the coordinates of the point where the filter is applied, $\boldsymbol{\xi}$ is the variable of spatial integration, and $G(\boldsymbol{x}-\boldsymbol{\xi})$ is the Gaussian spatial filter. As the eddy size reduces, so does the eddy viscosity within the LES framework. The Smagorinsky model[2] is utilized to approximate eddy viscosity across various turbulent scales, as demonstrated in the following expressions:

$$\tau_{ij}^{sgs}=-\frac{\partial}{\partial x_j}\rho\widehat{u_i' u_j'}=\mu_t\left[\frac{\partial \hat{u}_i}{\partial x_j}+\frac{\partial \hat{u}_j}{\partial x_i}\right] \tag{13}$$

$$\mu_t=\rho(C_s\Delta)^2\cdot\hat{S} \tag{14}$$

In this context, u_i' and u_j' represents the velocity profile of sub-grid eddies, μ_t denotes the corresponding dynamic viscosity, Δ represents the sub-grid length scale, C_s represents the Smagorinsky constant, and $\hat{S}$ stands for the resolved strain rate. In this sense, u_i' is not resolved in the solver. Instead, the complex interactions of these sub-grid eddies, typically expressed by the term $\frac{\partial}{\partial x_j}\rho\widehat{u_i' u_j'}$ is constitutively replaced

with a viscous stress tensor τ_{ij}^{sgs} to compensate the energy loss due to the cut-off of u_i' term (i.e., spatial filtering).

The comparison between LES and SST computational models against experimental benchmarks, depicted in Supplementary Fig. 10, indicates a minor difference in thrust predictions, peaking at 0.44 gf (0.8 %) at 5000 RPM. Torque predictions, however, diverge more, with errors increasing alongside rotational speeds. At 5000 RPM, the SST model's highest error registers at 0.00092 Nm (10.0 %), contrasted by the LES model's more modest 0.00032 Nm (3.5 %) discrepancy. Overall, LES provides a closer approximation of the propeller's aerodynamic performance.

Supplementary Table 1. Reynolds number associated with the propeller dynamics at various rotational speeds.

ω (RPM)	Mean Re	Max Re	Std Re
2000	7.499E+03	1.432E+04	5.963E+03
3000	1.125E+04	2.148E+04	8.944E+03
4000	1.500E+04	2.864E+04	1.193E+04
5000	1.875E+04	3.580E+04	1.491E+04
6000	2.250E+04	4.296E+04	1.789E+04

Supplementary figures

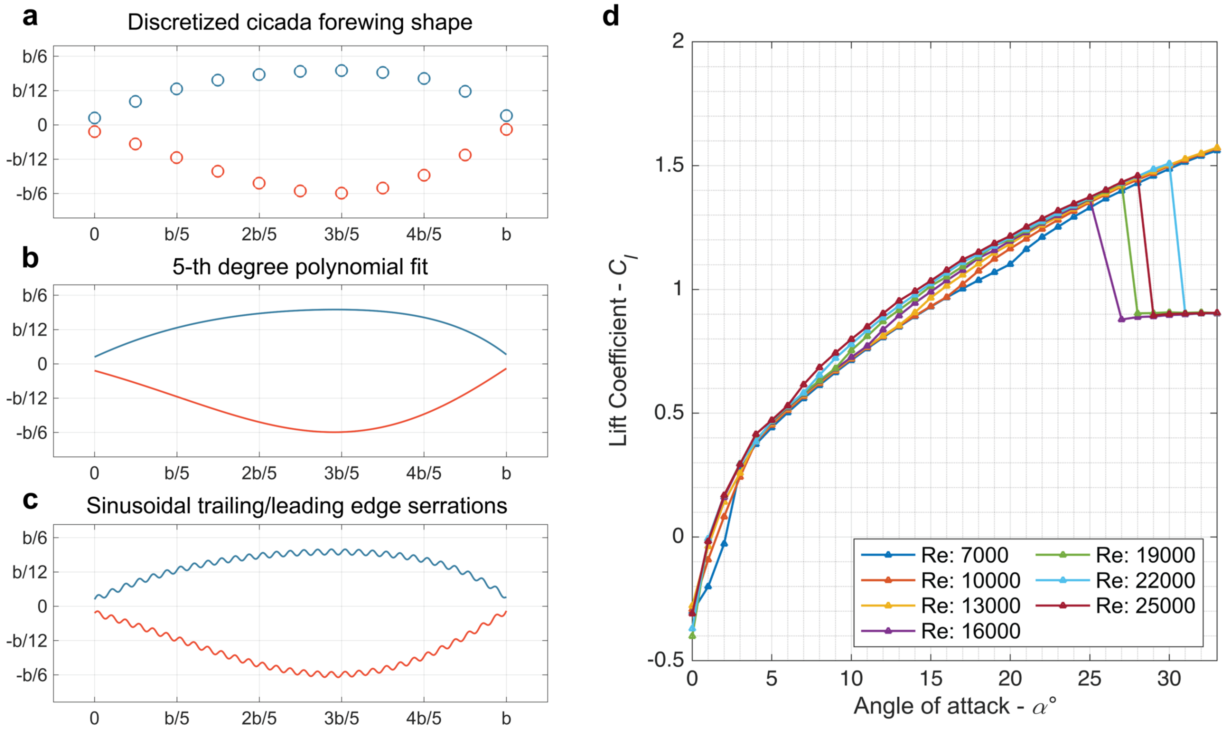

Supplementary Fig. 1. Illustration of 3D-SC planform concept and 2D airfoil stall. a Discrete points extracted from the outline of a cicada wing shape. **b** Fitted 5th-order polynomial curves for the approximation of leading and trailing edges of the wing. **c** Integration of a sinusoidal wave pattern into the wing planform. **d** Graph of the lift coefficient for a NACA 8412 airfoil profile versus angle of attack, demonstrating performance at varying *Re* numbers.

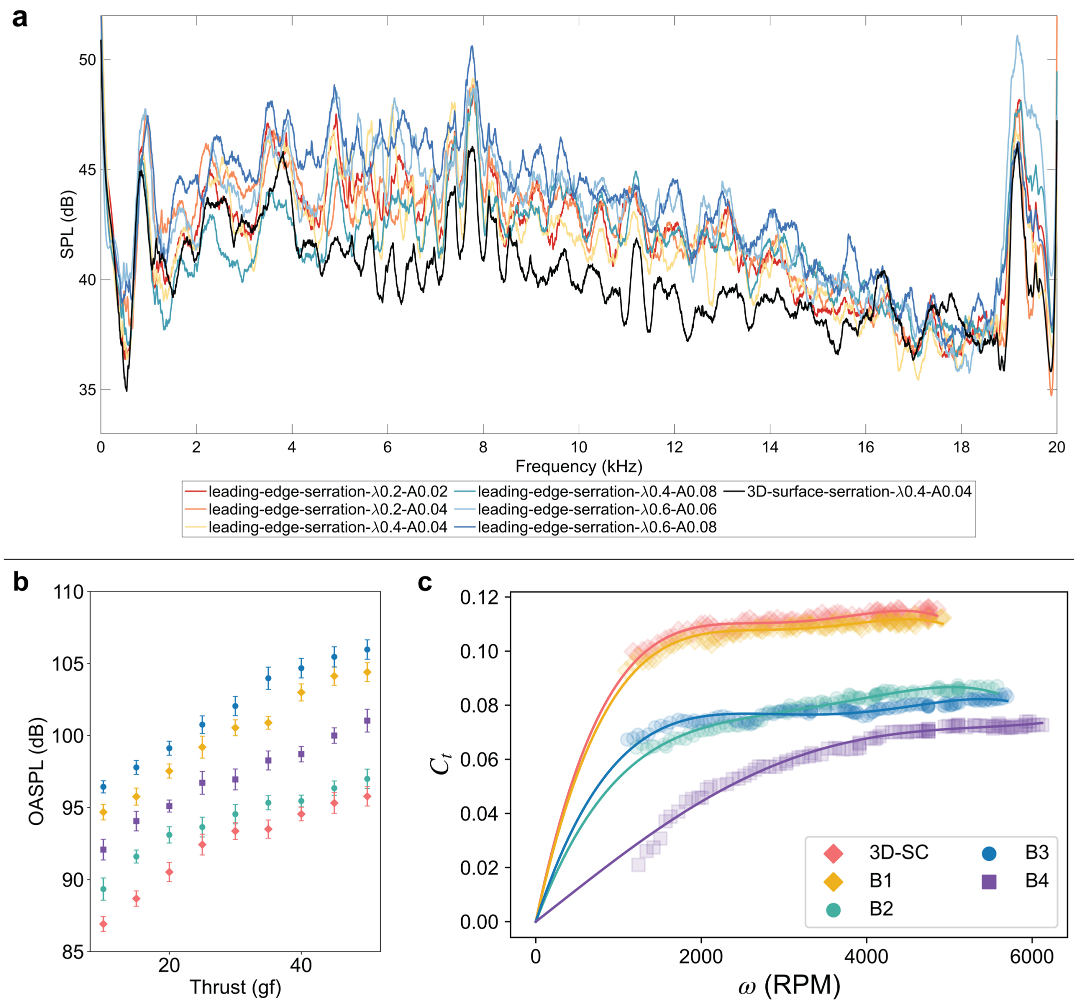

Supplementary Fig. 2. Supplementary experimental results. **a** Acoustic signature comparison using sound spectrum plots for propellers with 3D surface serration and 2D leading-edge serration. **b** Variation of A-weighted OASPL with thrust at a 5-meter measurement distance with error bars denoting one standard deviation of measurement variability. Each marker is positioned at the statistical mean of the corresponding data set. **c** Relationship between thrust coefficient and propeller rotational speed under test conditions.

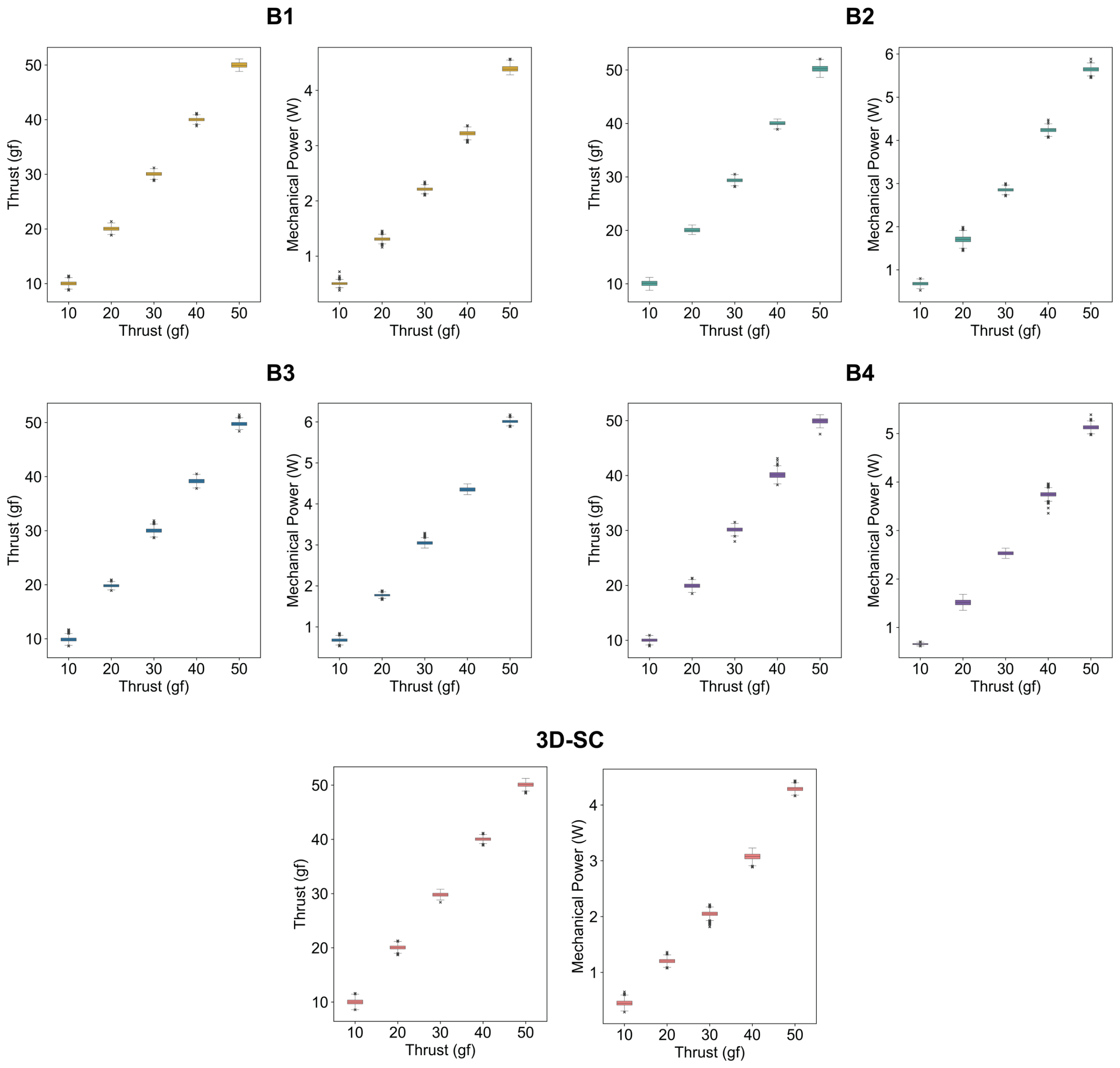

Supplementary Fig. 3. Quantification of measurement uncertainty. Boxplot visualization of thrust and mechanical power for different propeller prototypes across tested thrust levels. The plots synthesize 1000 data points each, collected at a consistent sampling frequency of 40 Hz. Error bars denote one standard deviation of measurement variability.

a

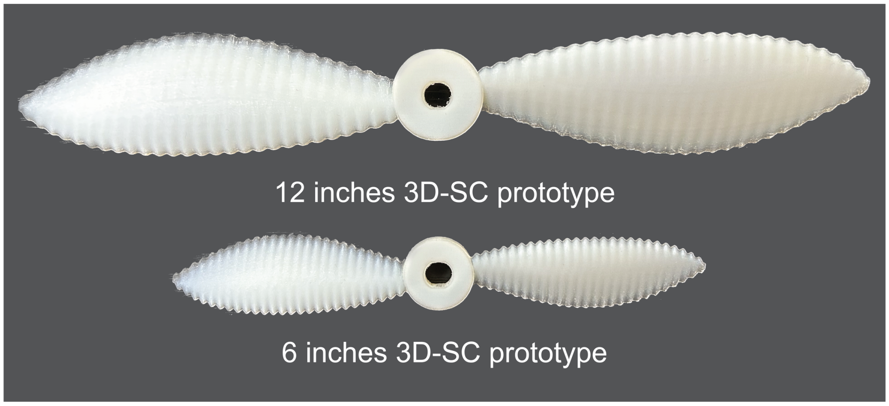

b

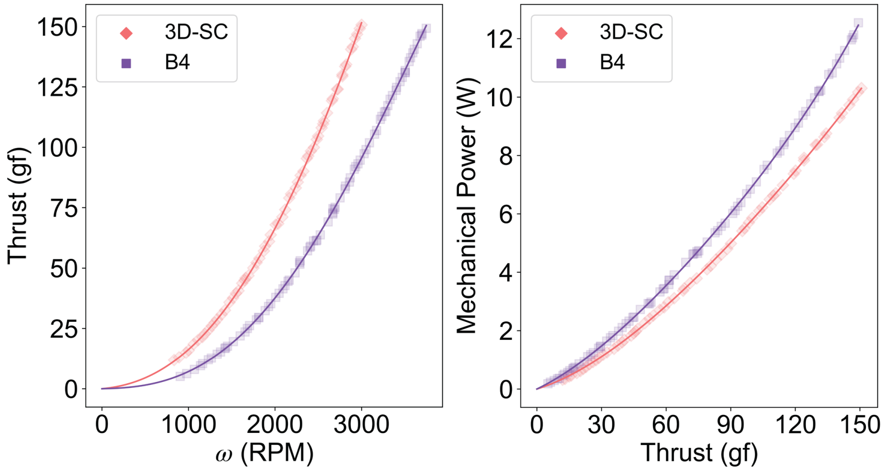

Supplementary Fig. 4. Aerodynamic characterization of 12-inch propeller prototypes. a Display of the 3D-printed prototypes with the 12-inch 3D-SC model situated above the 6-inch version. **b** Graphs presenting the relationship of thrust to rotational speed (left), and mechanical power versus thrust (right) for both the 12-inch 3D-SC and B4 prototypes.

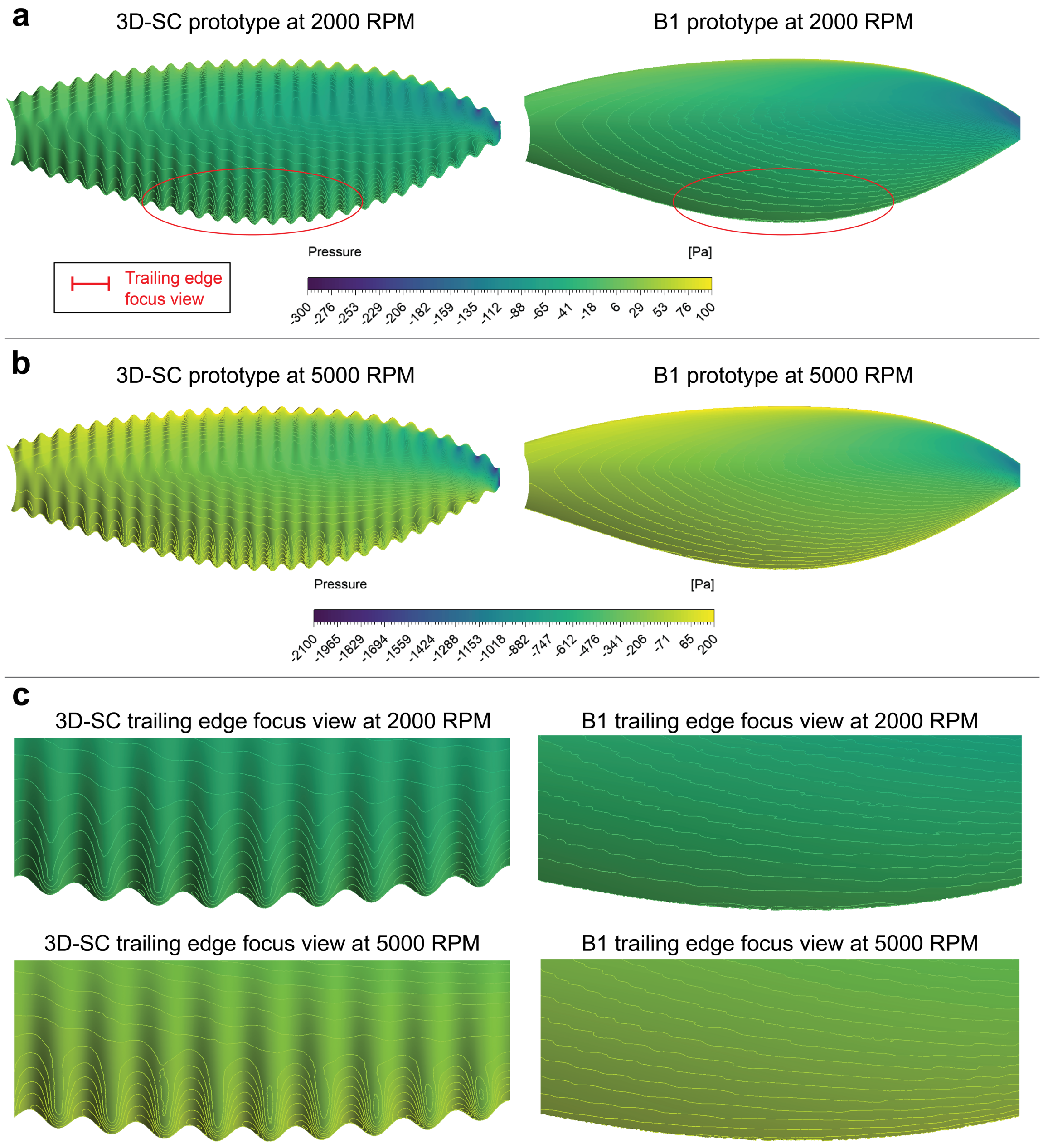

Supplementary Fig. 5. CFD pressure contour analysis on propeller surfaces. a Surface gauge pressure contours for 3DSC (left) and B1 (right) prototype models at 2000 RPM. **b** Surface gauge pressure contours at 5000 RPM. **c** This figure provides a focused view of the variations in pressure distribution patterns across the propeller surfaces of the 3D-SC and B1 prototypes at both 2000 and 5000 RPM.

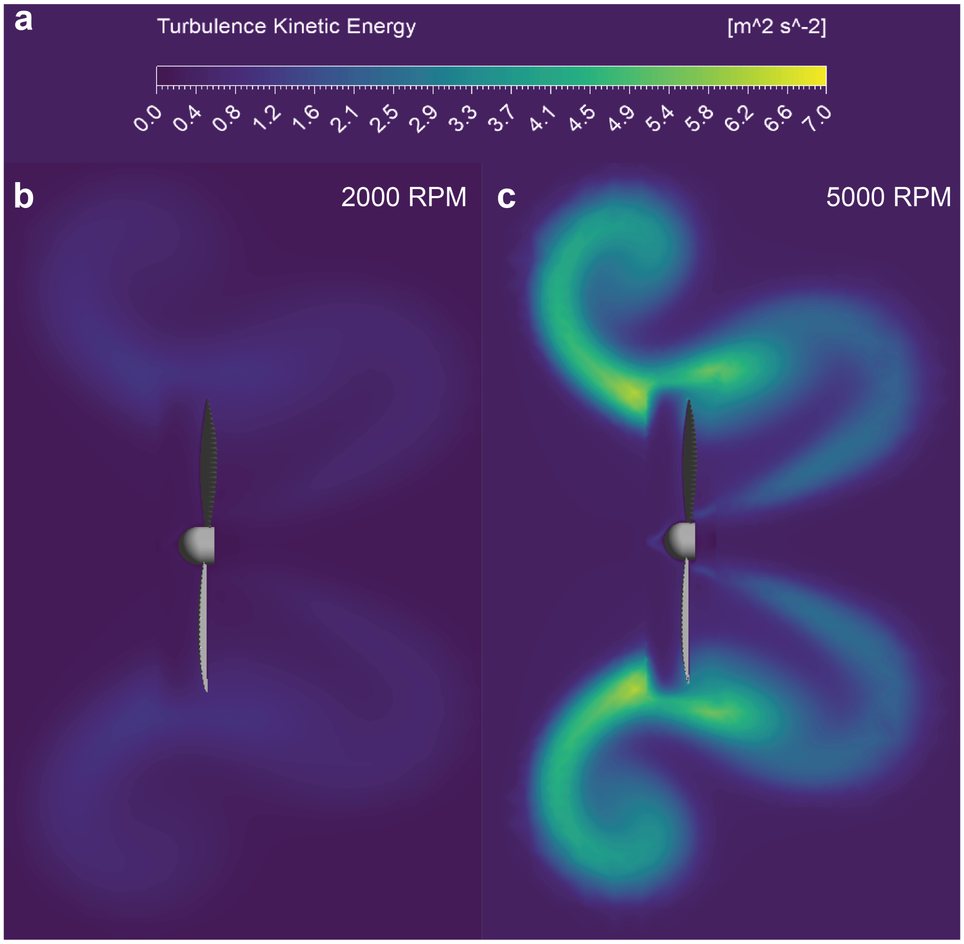

Supplementary Fig. 6. CFD turbulence kinetic energy contour. **a** Contour legend that corresponds to the turbulent kinetic energy level. **b** Turbulence kinetic energy contour of the 3D-SC propeller simulated at 2000 RPM. **c** Turbulence kinetic energy contour of the 3D-SC propeller simulated at 5000 RPM.

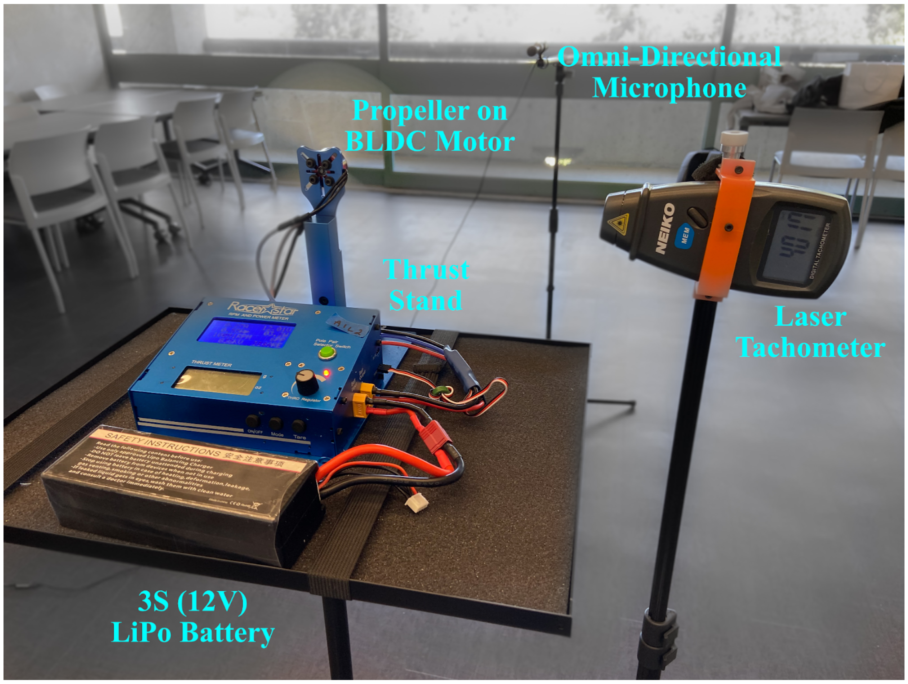

Supplementary Fig. 7. Experimental setup for sound data collection. The setup is comprised of various components: a NEIKO 20713A digital tachometer for measuring rotational speed; a Racerstar Motor Thrust Stand V3 utilized to secure and operate the propeller; a Readytosky 2212 920KV Brushless Motor, affixed to the thrust stand; an HRB 6000 mAh 3S Lithium Polymer (LiPo) Battery providing the necessary power; and a miniDSP UMIK-1 omnidirectional USB microphone for the capture of sound data.

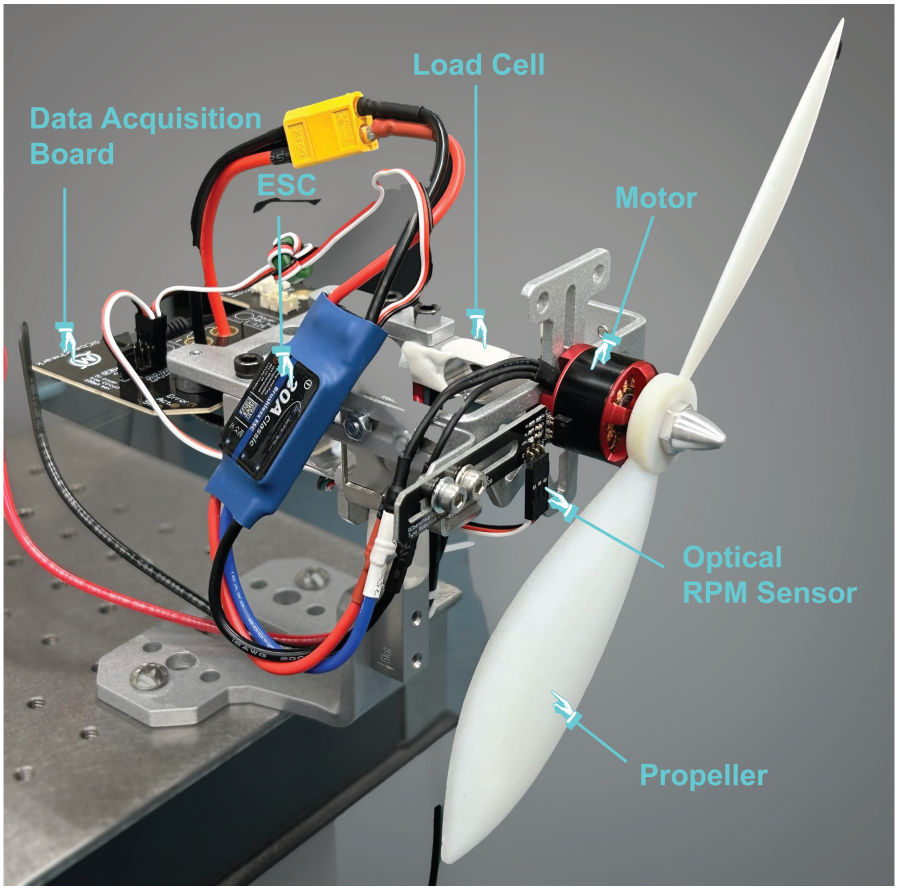

Supplementary Fig. 8. Experimental setup for aerodynamic data acquisition. The setup features a TYTO Series 1585 thrust stand, equipped with a TYTO optical RPM sensor for measuring rotational speed, load cells to detect thrust and torque, a 30-ampere brushless motor electronic speed controller for managing propeller speed, and a data acquisition board designed for on-the-fly data gathering.

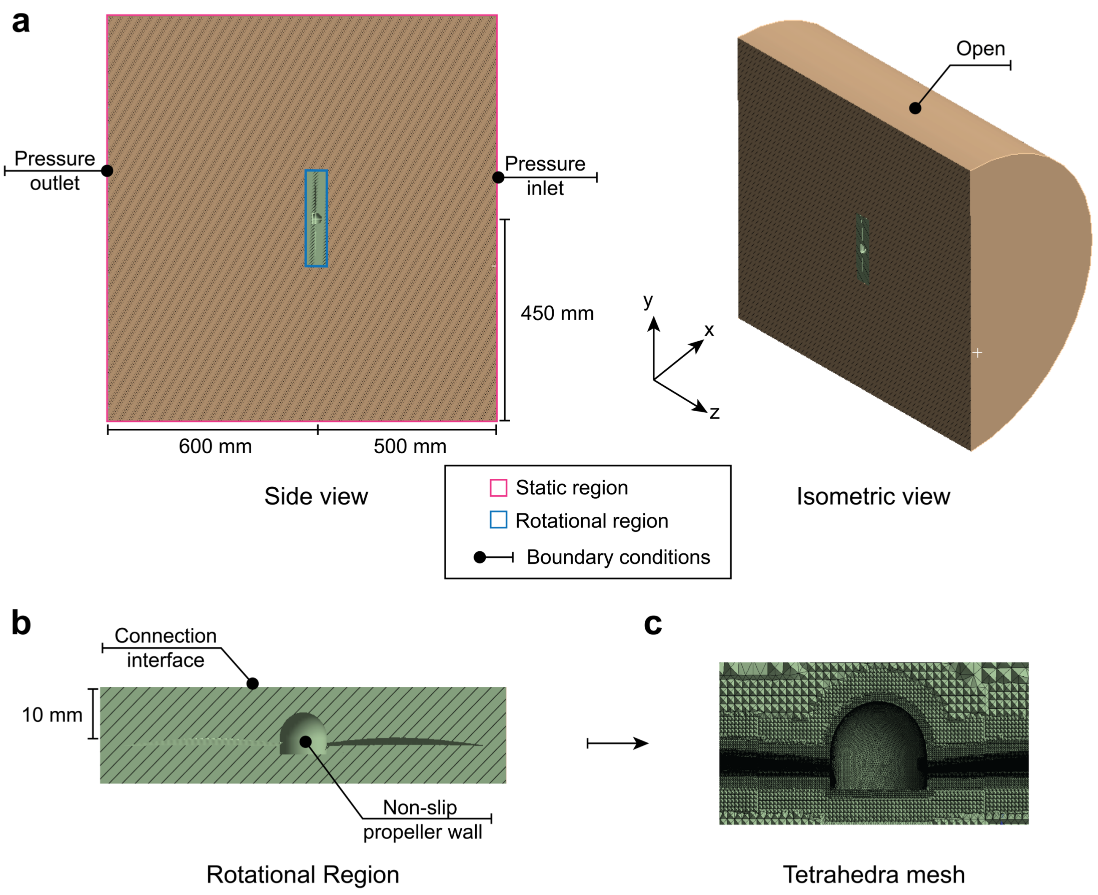

Supplementary Fig. 9. Fluid domain setup in CFD. a Illustration of the complete fluid domain employed in simulations, featuring a pressure inlet and outlet, along with an open cylindrical boundary. The static and rotational regions are delineated by red and blue boxes, respectively, with dimensions specified. **b** Detailed rotational region view, bordered by connection interfaces and a buffer zone measuring 10mm in height. **c** Visualization of the near-wall tetrahedral mesh constructed with high resolution.

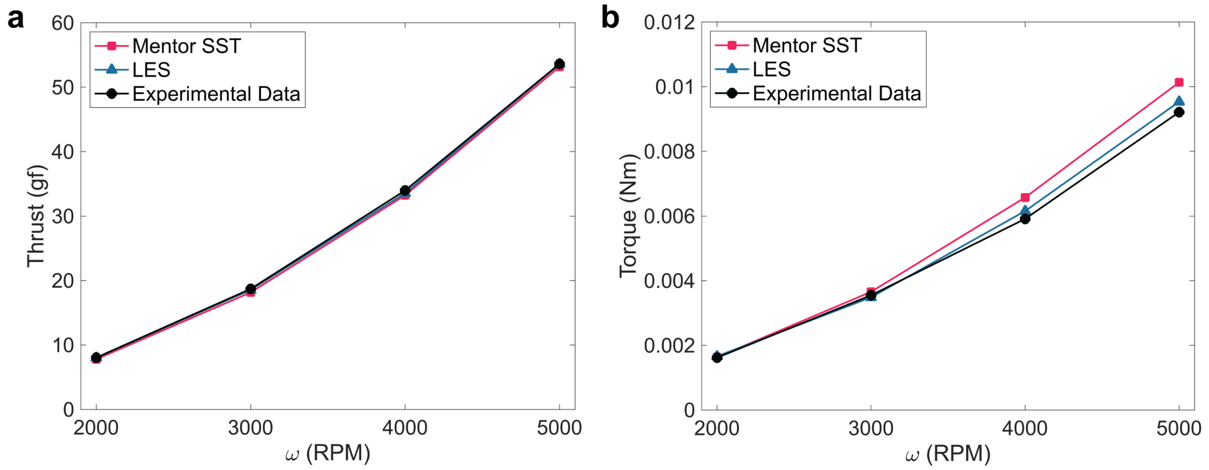

Supplementary Fig. 10. Validation of CFD turbulence models. a Comparative analysis of the computed propeller thrust using SST and LES turbulence models against measured data over a range of rotational speeds. **b** Comparative analysis of the computed propeller torque from SST and LES turbulence models as opposed to experimental data, across the same range of rotational speeds.

Supplementary References

1 Morfey, C. & Wright, M. Extensions of Lighthill's acoustic analogy with application to computational aeroacoustics. *Proceedings of the Royal Society A: Mathematical, Physical and Engineering Sciences* **463**, 2101-2127 (2007).

2 Smagorinsky, J. General circulation experiments with the primitive equations: I. The basic experiment. *Monthly weather review* **91**, 99-164 (1963).
